# Health literacy and health communication

**DOI:** 10.1186/1751-0759-4-18

**Published:** 2010-11-05

**Authors:** Hirono Ishikawa, Takahiro Kiuchi

**Affiliations:** 1 Department of Health Communication, Graduate School of Medicine, The University of Tokyo, 7-3-1 Hongo, Bunkyo, Tokyo, Japan

## Abstract

Health communication consists of interpersonal or mass communication activities focused on improving the health of individuals and populations. Skills in understanding and applying information about health issues are critical to this process and may have a substantial impact on health behaviors and health outcomes. These skills have recently been conceptualized in terms of health literacy (HL). This article introduces current concepts and measurements of HL, and discusses the role of HL in health communication, as well as future research directions in this domain. Studies of HL have increased dramatically during the past few years, but a gap between the conceptual definition of HL and its application remains. None of the existing instruments appears to completely measure the concept of HL. In particular, studies on communication/interaction and HL remain limited. Furthermore, HL should be considered not only in terms of the characteristics of individuals, but also in terms of the interactional processes between individuals and their health and social environments. Improved HL may enhance the ability and motivation of individuals to find solutions to both personal and public health problems, and these skills could be used to address various health problems throughout life. The process underpinning HL involves empowerment, one of the major goals of health communication.

## Introduction

Health communication, i.e., interpersonal or mass communication activities focused on improving the health of individuals and populations [1], has emerged as one of the most important public health issues in this century. The Healthy People 2010 project in the US suggests that health communication can contribute to all aspects of disease prevention and health promotion and that it is relevant to a number of domains including (1) health professional-patient relations, (2) individuals' exposure to, search for, and use of health information, (3) individuals' adherence to clinical recommendations and regimens, (4) construction of public health messages and campaigns, (5) dissemination of individual and population health risk information, that is, risk communication, (6) images of health in the mass media and the culture at large, (7) education of consumers about how to gain access to the public health and health care systems, and (8) development of telehealth applications.

People in modern society are expected to actively engage in the management of their health and to make a wide range of health decisions. Sound health decisions require comprehensible health information that is accessible and appropriate to the needs and cultural and social backgrounds of individuals [2]. Although health care professionals have historically been the primary sources of health and medical information, the increase in media reports and the rapid expansion of the Internet have rendered other sources more available to the general public [3-6]. Thus, skills in understanding and applying information about health issues may have a substantial impact on health behaviors and health outcomes. These skills have recently been conceptualized as health literacy (HL).

One of the objectives related to health communication in the US Healthy People 2010 project involves improving the HL of persons with inadequate or marginal literacy skills. Indeed, significant concern that people with limited HL may not be able to adequately understand health information, even in the presence of access to such information and related services, has emerged. In some cases, more information may actually cause feelings of confusion and powerlessness instead of facilitating sound health decisions. Even when health information is not intentionally sought, it may be provided by the media or by anyone with whom individuals communicate. The need for improved HL has become apparent as the number of health information sources that are easily accessed by the general public has increased in the absence of established assurances of the quality of the information provided by such sources.

The purpose of this paper is to introduce currently used concepts and measures of HL and to present an example of a study of HL in Japan. We then discuss the role of HL in health communication and outline a future research agenda for this domain.

### The concept of health literacy

In general, literacy is the ability to read, write, and speak a language in the service of understanding and solving problems with sufficient proficiency to function at work and in society, achieve goals, and develop knowledge and individual potential (US Congress, National Literacy Act of 1991, Public Law 102-73, 1991). The notion of HL is based on this concept of literacy and generally refers to literacy in the context of health and healthcare. Given that basic literacy skills are required for health literacy, it is reasonable to assume that individuals with limited literacy also have limited HL. Indeed, previous studies have reported significant associations between measures of literacy and measures of functional HL, such as the Rapid Estimate of Adult Literacy in Medicine (REALM) [7]and the Test of Functional Health Literacy in Adults (TOFHLA) [8]. On the other hand, it has been noted that even individuals with adequate general literacy might not have adequate HL because the literacy demands in the context of healthcare are frequently more complex than those in the context of everyday life [9].

Several definitions of HL are currently used; these share the basic concept of literacy, but vary in scope. The US Healthy People 2010 project refers to HL as "the degree to which individuals have the capacity to obtain, process, and understand basic health information and services needed to make appropriate health decisions" [10]. Compared with earlier definitions of HL that focus on patients in healthcare settings and their understanding of medical information, this definition includes individuals outside of clinical settings and also links health literacy to the promotion of health and preventive behaviors.

Another well-recognized definition, proposed by the World Health Organization (WHO), defines HL more broadly as "the cognitive and social skills which determine the motivation and ability of individuals to gain access to, understand, and use information in ways which promote and maintain good health" [1]. This version also suggests that HL entails a level of knowledge, personal skills, and confidence that enables making changes in personal lifestyles and living conditions to improve personal and community health. Thus, this definition includes issues critical to the empowerment of patients. It also focuses not only on the cognitive elements of comprehending, analyzing, and applying health information to decisions about health, but also on the social skills involved in those interactions with other people and society (e.g., communication, negotiation, and organization) that are necessary for transforming decisions into practice. This conceptualization also refers to *motivation *in addition to *ability*. Based on this conceptualization, Nutbeam [11] proposed a model of HL that includes three levels and assumes the existence of benefits to both the *individual *and the *population *at each level: (1) basic/functional literacy, including skills for reading and writing that enable effective functioning in everyday situations, which is broadly compatible with the narrow definition of HL; (2) communicative/interactive literacy, including more advanced skills that enable active participation in everyday activities, extracting and understanding information from different sources, and applying new information to changing circumstances; and (3) critical literacy, including more advanced skills for critically analyzing information and using this information to exert greater control over life events and situations.

As the field of HL has expanded in scope and depth, the term HL itself has come to have different meanings. Nutbeam [12] distinguished two different approaches to HL: HL as clinical "risk" and HL as personal "asset." According to the former, HL is considered to be a set of individual literacy skills that act as a mediating factor in health and clinical decision making. The definition of HL according to the US Healthy People 2010 project is linked to this conceptualization. In contrast, the conceptualization of HL as a personal asset has evolved from public health and health promotion perspectives. In this context, HL is a means for enabling individuals to exert greater control over their health as well as over the range of personal, social, and environmental determinants of health; this corresponds to the definition issued by WHO. A similar distinction has been proposed by Peerson and Saunders [13]: "medical literacy," which is related to individuals as patients within health care settings, versus "health literacy," which is related to everyday life.

### Measurement of health literacy

In general, literacy includes a variety of skills beyond reading and writing, such as numeracy, listening, and speaking, and relies on cultural and conceptual knowledge [9]. Nonetheless, most existing measures of HL have focused primarily on reading comprehension and numeracy. Typically, the REALM, the TOFHLA, and their short versions have been used in the US in clinical situations as screening tools to identify patients with limited HL.

A recent review identified 19 instruments for measuring HL that were published between 1990 and 2008 and examined their content and psychometric properties [14]. These included instruments that directly test an individual's abilities (e.g., the REALM, the TOFHLA, the Newest Vital Sign [15]), self-reports of abilities (e.g., Functional, Communicative, and Critical Health Literacy Scales [16], the Set of Brief Screening Questions [17]), and population-based proxy measures (e.g., the National Assessment of Adult Literacy [18], and the Health Activities Literacy Scale [19]). They concluded that the composition of the underlying constructs and the nature of the content varied widely across HL instruments, rendering it difficult to interpret and compare studies.

It has also been noted that none of the existing instruments appears to completely measure the concept of HL as defined in the previous section. In particular, measurements of communicative/interactive and critical HL have lagged far behind instruments addressing functional HL [20]. Much work remains in the effort to develop more comprehensive measures that will assess individual HL with respect to an individual's ability to access, understand, and use health information in ways that promote and maintain good health [12]. Although a few recent studies have developed self-report measures of communicative and critical HL and examined their impact on health behaviors and outcomes [16,21], the development of an objective and direct measure for communicative and critical HL may pose a greater challenge than the development of such a measure for functional HL, such as the REALM and the TOFHLA. Skills relevant to clinical encounters may be assessed with a coding system applied to recorded communication between patient and healthcare providers, such as the Roter Interaction Analysis Systems [22], whereas skills relevant to other settings, such as those in which information obtained from the mass media or Internet is sought or used, may be more difficult to assess. Assessment difficulties also derive from the fact that the skills necessary will vary depending on the demands placed on the patient by the environment, including healthcare providers, healthcare systems, and the media. Thus, an HL level that is "adequate" in one situation may be inadequate in another situation, and this context dependence is especially true for communicative and critical HL.

In this sense, it is likely that different measurement tools will be required for measuring HL in different contexts [23]. Although previous instruments have approached HL as a quality characterizing the individual, HL is now seen as also based on interactions between an individual's skills and the demands of the society in which the individual lives, including healthcare providers, the healthcare system, the media, and the community [9,24]. Thus, an individual's HL should be defined and assessed in relation to the ability of the society to communicate health information in a manner appropriate to the audience (i.e., the HL of the population). However, further difficulties may arise in the development of an objective measurement of this type of HL [24].

### A health literacy study in Japan

In our previous study, we developed self-rating scales measuring the functional, communicative, and critical HL of patients with chronic diseases [16]. Item content and mean scores from this study are shown in Table 1. Functional HL was assessed with five items that examined the extent to which patients experienced difficulties in reading the instructions or leaflets provided by hospitals and pharmacies (Cronbach's *α *= 0.84). Communicative HL was evaluated with five items that assessed the extent to which patients extracted and communicated diabetes-related information since they were diagnosed with this disease (*α *= 0.77). Critical HL was assessed with four items that focused on the extent to which patients had critically analyzed diabetes-related information and used it to make decisions (*α *= 0.65). Higher scores on this HL scale were generally associated with better knowledge of diabetes, a greater number of information sources, and higher self-efficacy with respect to diabetes self-care. Furthermore, patient HL, especially communicative HL, was related to the process of communicating with physicians during medical visits [25]. Moving beyond previous measures focusing solely on functional HL, this HL scale included three levels of HL, each of which may reflect different effects on health behaviors and outcomes. This measure also proved to be easy to administer in a clinical setting.

**Table 1:** Item content and means of the Functional, Communicative and Critical HL scales

	Mean	SD
**Functional health literacy**	**3.39**	**0.75**
In reading instructions or leaflets from hospitals/pharmacies, you....		
found that the print was too small to read	3.19	1.12
found characters and words that you did not know	3.41	0.88
found that the content was too difficult	3.43	0.84
needed a long time to read and understand them	3.27	1.04
needed someone to help you read them	3.65	0.86
**Communicative health literacy**	**2.56**	**0.70**
Since being diagnosed with diabetes, you have....		
collected information from various sources	2.43	1.04
extracted the information you wanted	2.18	1.00
understood the obtained information	2.89	0.88
communicated your thoughts about your illness to someone	2.70	0.91
applied the obtained information to your daily life	2.60	0.99
**Critical health literacy**	**1.96**	**0.63**
Since being diagnosed with diabetes, you have....		
considered whether the information was applicable to your situation	2.71	0.98
considered the credibility of the information	1.87	0.92
checked whether the information was valid and reliable	1.76	0.96
collected information to make health-related decisions	1.51	0.77

Note: The theoretical range: 1-4.

Based on this study among diabetes patients, a short version of the communicative and critical HL scale for general populations was validated in our study of office workers [21]. Item content and mean scores are shown in Table 2. In our analyses, higher HL was associated with healthy lifestyles and more effective coping with job stress as well as with fewer somatic symptoms.

**Table 2:** Item content and means of the Communicative and Critical HL scales

	Mean	SD
1) Seeking information from various sources	4.13	0.80
2) Extracting relevant information	3.92	0.82
3) Understanding and communicating the information	3.56	0.85
4) Considering the credibility of the information	3.52	0.89
5) Making decisions based on the information	3.42	0.95
Total scale score (Mean, SD)	3.72	0.68

Note: The theoretical range: 1-5.

One of the limitations of these studies was that we were unable to examine the relationship between our new HL scales and the existing standard measures of functional HL, such as the TOFHLA or REALM, because they were not available in Japanese at the time of our study. We noted that this issue should be explored in a future study with an English-speaking population to further revise and validate our HL scales in the service of enhancing their utility. After publication of these articles, several researchers in the US, Australia, the Netherlands, and Germany contacted us for validation of the HL scales in their countries.

## Conclusions

Although studies of HL have increased dramatically during the past few years, a gap between the conceptual definition of HL and its application remains. More specifically, studies on communicative/interactive and critical HL are particularly limited. Furthermore, HL should be considered not only as a characteristic of an individual, but also as a feature of interactions involving an individual's HL and his/her health and social environments.

In contrast to the high-risk approach adopted in traditional health education, which seeks to protect susceptible individuals [26] through such actions as screening those at high risk during health checkups to provide health counseling, the concept of HL may facilitate the development of a population-based approach. Such an approach would seek to control the causes of health problems, including eliminating the barriers that prevent individuals with limited HL from participating in the healthcare process and improving the HL of the population as a whole. Previous HL intervention programs have frequently attempted to decrease specific barriers affecting those with limited HL, including teaching healthcare providers to better communicate with patients with limited HL or developing simple and attractive health education materials pitched at those with lower reading levels [27-29]. Future interventions should be expanded to include methods for improving popular HL through school-based health education directed at children and adolescents as well as through more general efforts directed at adults. Improved HL could enhance the ability and motivation of individuals to solve personal and public health problems by enabling them to apply skills in response to various health problems arising throughout life. This process of empowerment constitutes one of the major goals of health communication.

## Competing interests

The authors declare that they have no competing interests.

## Authors' contributions

HI wrote the first draft of the paper, and TK revised it critically for important intellectual content. Both authors hold final responsibility for the decision to submit the paper for publication.

## References

[B1] Nutbeam D Health promotion glossary Health Promot Int 1998 13 349 364 10.1093/heapro/13.4.349

[B2] Kickbusch I Maag D Heggenhougen K, Quah S Health literacy Encyclopedia of public health 2008 3 San Diego: Academic Press 204 211 full_text

[B3] Hesse BW Nelson DE Kreps GL Croyle RT Arora NK Rimer BK Viswanath K Trust and sources of health information: the impact of the Internet and its implications for health care providers: findings from the first Health Information National Trends Survey Arch Intern Med 2005 165 2618 2624 10.1001/archinte.165.22.2618 16344419

[B4] Napoli PM Rice RE, Katz JE Consumer use of medical information from electronic and paper media The internet and health communication: Experiences and expectations 2001 Thousand Oaks, CA: Sage Publications, Inc 79 98

[B5] Passalacqua R Caminiti C Salvagni S Barni S Beretta GD Carlini P Contu A Di Costanzo F Toscano L Campione F Effects of media information on cancer patients' opinions, feelings, decision-making process and physician-patient communication Cancer 2004 100 1077 1084 10.1002/cncr.20050 14983505

[B6] Rutten LJ Arora NK Bakos AD Aziz N Rowland J Information needs and sources of information among cancer patients: a systematic review of research (1980-2003) Patient Educ Couns 2005 57 250 261 10.1016/j.pec.2004.06.006 15893206

[B7] Davis TC Long SW Jackson RH Mayeaux EJ George RB Murphy PW Crouch MA Rapid estimate of adult literacy in medicine: a shortened screening instrument Fam Med 1993 25 391 395 8349060

[B8] Parker RM Baker DW Williams MV Nurss JR The test of functional health literacy in adults: a new instrument for measuring patients' literacy skills J Gen Intern Med 1995 10 537 541 10.1007/BF02640361 8576769

[B9] Nielsen-Bohlman L Panzer AM Kindig DA Health literacy: A prescription to end confusion 2004 Washington, DC: The National Academies Press 25009856

[B10] U.S Department of Health and Human Services Healthy People 2010: Understanding and Improving Health 2000 2 Washington, DC: Government Printing Office

[B11] Nutbeam D Health literacy as a public health goal: a challenge for contemporary health education and communication strategies into the 21st century Health Promot Int 2000 15 259 267 10.1093/heapro/15.3.259

[B12] Nutbeam D The evolving concept of health literacy Soc Sci Med 2008 67 2072 2078 10.1016/j.socscimed.2008.09.050 18952344

[B13] Peerson A Saunders M Health literacy revisited: what do we mean and why does it matter? Health Promot Int 2009 24 285 296 10.1093/heapro/dap014 19372101

[B14] Jordan JE Osborne RH Buchbinder R J Clin Epidemiol 2010 20638235 10.1016/j.jclinepi.2010.04.005

[B15] Weiss BD Mays MZ Martz W Castro KM DeWalt DA Pignone MP Mockbee J Hale FA Quick assessment of literacy in primary care: the newest vital sign Ann Fam Med 2005 3 514 522 10.1370/afm.405 16338915 PMC1466931

[B16] Ishikawa H Takeuchi T Yano E Measuring functional, communicative, and critical health literacy among diabetic patients Diabetes Care 2008 31 874 879 10.2337/dc07-1932 18299446

[B17] Chew LD Bradley KA Boyko EJ Brief questions to identify patients with inadequate health literacy Fam Med 2004 36 588 594 15343421

[B18] Kutner M Greenberg E Jin Y Paulsen C The Health Literacy of America's Adults: Results From the 2003 National Assessment of Adult Literacy (NCES 2006-483) 2006 Washington, DC: National Center for Education Statistics

[B19] Rudd RE Health literacy skills of U.S. adults Am J Health Behav 2007 31 Suppl 1 S8 18 17931141 10.5555/ajhb.2007.31.supp.S8

[B20] Ishikawa H Yano E Patient health literacy and participation in the health-care process Health Expect 2008 11 113 122 10.1111/j.1369-7625.2008.00497.x 18494956 PMC5060442

[B21] Ishikawa H Nomura K Sato M Yano E Developing a measure of communicative and critical health literacy: a pilot study of Japanese office workers Health Promot Int 2008 23 269 274 10.1093/heapro/dan017 18515303

[B22] Roter D Larson S The Roter interaction analysis system (RIAS): utility and flexibility for analysis of medical interactions Patient Educ Couns 2002 46 243 251 10.1016/S0738-3991(02)00012-5 11932123

[B23] Nutbeam D Defining and measuring health literacy: what can we learn from literacy studies? Int J Public Health 2009 54 303 305 10.1007/s00038-009-0050-x 19641847

[B24] Baker DW The meaning and the measure of health literacy J Gen Intern Med 2006 21 878 883 10.1111/j.1525-1497.2006.00540.x 16881951 PMC1831571

[B25] Ishikawa H Yano E Fujimori S Kinoshita M Yamanouchi T Yoshikawa M Yamazaki Y Teramoto T Patient health literacy and patient-physician information exchange during a visit Fam Pract 2009 26 517 523 10.1093/fampra/cmp060 19812242

[B26] Rose G Sick individuals and sick populations Int J Epidemiol 1985 14 32 38 10.1093/ije/14.1.32 3872850

[B27] Gerber BS Brodsky IG Lawless KA Smolin LI Arozullah AM Smith EV Berbaum ML Heckerling PS Eiser AR Implementation and evaluation of a low-literacy diabetes education computer multimedia application Diabetes Care 2005 28 1574 1580 10.2337/diacare.28.7.1574 15983303

[B28] Kalichman SC Cherry J Cain D Nurse-delivered antiretroviral treatment adherence intervention for people with low literacy skills and living with HIV/AIDS J Assoc Nurses AIDS Care 2005 16 3 15 16433105 10.1016/j.jana.2005.07.001

[B29] van Servellen G Nyamathi A Carpio F Pearce D Garcia-Teague L Herrera G Lombardi E Effects of a treatment adherence enhancement program on health literacy, patient-provider relationships, and adherence to HAART among low-income HIV-positive Spanish-speaking Latinos AIDS Patient Care STDS 2005 19 745 759 10.1089/apc.2005.19.745 16283835

